# Highlighting the results of a trial by using appropriate inferential statistics: Commentary on “Specific exercises for subacromial pain” by Björnsson Hallgren et al. 2017

**DOI:** 10.1080/17453674.2018.1441965

**Published:** 2018-04-05

*Sir,—*Subacromial pain is a very common cause of visits to an orthopedic clinic. We have read with interest the paper “Specific exercises for subacromial pain” by Björnsson-Hallgren et al. The question whether an exercise program specifically developed to treat subacromial pain is superior to a regular training regime is important. Recognizing the strengths of the study, we would like to discuss some issues concerning the approach used by the authors when reporting the results. The main results were reported as absolute numbers of exposed and unexposed patients in each group along with p-values. The exposure was a decision to agree to a surgical procedure at the end of follow-up. Such an approach may create a problem: absolute estimates describe a particular single sample but say nothing about the entire population of interest. Based on the reported results, we discover that 14 of 47 patients in the intervention group and 28 of 44 controls ended in a decision to undergo surgery. Additionally, we find out that the difference between these two particular groups was statically significant as shown by the p-value <0.05.

While such statistics are adequate to inform readers about certain differences between groups, several important questions remain unanswered:

- How substantial was the difference between groups—how much higher risk did controls have regarding the surgery decision?
- How wide could the possible boundaries of observed difference be if different random samples containing different individuals were drawn from the population of patients with subacromial pain?
- How many patients have to be treated by a specific training program in order to avoid at least one surgery decision?

The first question can be answered by calculating, e.g., a relative risk ratio (RR), the second by drawing a confidence interval (95% CI), and the third question can be answered by estimating a number needed to treat (NNT). These statistics can be calculated in Excel, or by the majority of statistical packages available on the market, or even by hand.

Thus, the following statistics may be added to the report:

- RR =0.47 (meaning that the intervention group had around half the risk of ending up with a decision for surgery when compared with controls);
- p-value of RR =0.0025 (the difference between the two groups regarding the risk was statistically significant);
- 95% CI for RR =0.27–0.77 (the risk ratio may vary within these limits if different patients are drawn from the population);
- NNT =3.0 (at least 3 patients need to be included in a specific exercise group in order to avoid 1 decision for surgery);
- 95% CI for NNT is 1.9–6.9 (the NNT will probably fall into these limits if different patients are drawn from the population).

In this way, the report may come to be more robust and precise, emphasizing substantially the conclusion made by the authors. We encourage researchers to report statistics that describe a population (“inferential statistics”) whenever possible.

**Mikhail Saltychev ^1^ and Petri Virolainen ^2^**

Departments of ^1^ Physical and Rehabilitation Medicine and ^2^ Orthopedics, Turku University Hospital and University of Turku, Turku, Finland

Email: mikhail.saltychev@gmail.com

*Sir,—We* thank M Saltychev and P Virolainen for their interest in our work. We appreciate their approach to the research question, which may provide an additional viewpoint and further demonstrates the differences between the study groups. We have controlled our data according to number needed to treat (NNT) and relative risk (RR) and confirmed that their calculations are accurate. These statistical results allow for a slightly different perspective on our results, which may be even clearer in presenting our results for the majority of readers treating subacromial pain patients. They further emphasize the importance of a specific exercise program and may very well be added to our results. We found their comments very helpful and valuable for our future research.

**Hanna Björnsson Hallgren,** corresponding author

Email: hanna.bjornsson.hallgren@regionostergotland.se

Björnsson Hallgren H C, Adolfsson L E, Johansson K, Öberg B, Peterson A, Holmgren T M. Specific exercises for subacromial pain: Good results maintained for 5 years. Acta Orthop 2017; 88 (6): 600-605.

